# Removing Educational Achievement Points From the Foundation Programme Application System: Is This the Right Decision?

**DOI:** 10.2196/27856

**Published:** 2021-08-04

**Authors:** Abirami Ganesh Kumar, Georgios Kallikas, Melihah Hassan, Indu Kiran Dev, Soutrik Basu

**Affiliations:** 1 Department of Medicine London School of Medicine and Dentistry Queen Mary University of London Barts Whitechapel, East London United Kingdom

**Keywords:** medical student, medical education, research, academic medicine, medical school, United Kingdom, achievement, test scores, transferable skills

## Abstract

The UK Foundation Programme Office has announced that medical students graduating from 2023 onward will not receive Foundation Programme Application System points for additional degrees or journal publications. In this viewpoint paper, we acknowledge the reasons for this decision, such as socioeconomically advantaged students having greater access to these achievements and the promotion of intercalated degrees for the sake of point accumulation. Additionally, the predictive value of these achievements with regard to junior doctors’ performance has been questioned when compared to that of other Foundation Programme Application System components. Conversely, we also highlight the drawbacks of the UK Foundation Programme Office’s decision, since this might discourage medical students from completing additional degrees and attempting to publish their work, thereby resulting in clinicians with little to no academic experience or interest. Finally, we attempt to provide suggestions for future improvements in this system by analyzing different medical schools’ approaches, such as the BMedSci Honors program offered at Nottingham University. Furthermore, promoting and supporting engagement with academia, especially among socioeconomically disadvantaged students, are the responsibility of all medical schools; such actions are needed in order to produce doctors who are both clinically and academically competent. We conclude that the aforementioned changes should only affect new cohorts in the interest of universities’ transparency and fairness to their students.

## Introduction

On November 30, 2020, the UK Foundation Programme Office (UKFPO) announced their decision to reform the points-based Foundation Programme Application System (FPAS) by removing educational achievement (EA) point scores. This reform is set to take effect in 2023 [1]. In this viewpoint paper, we aim to summarize the benefits and drawbacks of the changes proposed by the UKFPO as well as offer potential solutions to the issues presented by this reform.

The UKFPO is the governing body that manages the transition of graduating medical students into Foundation Programme rotations as newly qualified doctors by using the FPAS scoring system to allocate junior doctors to different locations [1]. The FPAS scoring system is a method of ranking final year medical students nationally by using a variety of parameters to award them with up to 100 points. The more FPAS points that an applicant has, the more likely they are to be accepted into their top choice rotation in their preferred deanery. The two main parameters used in the FPAS scoring system are the Situational Judgement Test and Educational Performance Measure (EPM), which have been used in equal proportion [2]. The EPM score is primarily calculated by using students’ performance at medical school in the form of decile rankings. EAs are optional components that also contribute to the EPM score and are achieved by completing additional degrees and having work published in PubMed-indexed journals.

Obtaining intercalated degrees is a popular choice among medical students; students can take 1 year off from their medical studies to explore other fields of interest, thereby gaining an additional degree and opportunities to present at conferences and publish their work [2]. Some students also obtain EA points by completing additional degrees prior to attending medical school. Further, EA points can be earned by publishing original research or other article types, such as letters to the editor, commentaries, and case studies.

## The Disadvantages of EA Points in the FPAS

We acknowledge the UKFPO’s reasoning that opportunities for completing EAs favor those from more advantaged backgrounds [3]. Obtaining an intercalated degree is costly in terms of tuition fees and living expenses, and graduating 1 year later can result in a delay of 1 year’s worth of earnings. These factors are more detrimental to students from lower socioeconomic backgrounds [4]. Moreover, the value of EA points that are achieved by publishing articles can be skewed by wealthier students paying article processing charges in order to publish their work more easily in lower-impact journals [5]. Similarly, some institutions cover the cost of article processing charges for their students. This is not standard practice across all medical schools and therefore creates nationwide inequalities in opportunities.

As highlighted by the UKFPO, in several UK medical schools, intercalated degrees are compulsory components that have been integrated into 6-year courses. As such, admission into these institutions will assuredly gain students EA points. Since intercalated degrees are closely linked to publication opportunities, they can further the advantages of mandatory intercalation [1]. In contrast, the number of students who are allowed to intercalate at universities where intercalation is optional is often limited [6]. This creates a biased system in which some students have advantages in gaining EA points depending on their medical schools. Additionally, medical students who have already obtained additional degrees prior to entering medical school are also advantaged; this cohort of students makes up approximately 8% of the medical student population [7]. As a result, students who hold additional degrees and those who attend institutions with compulsory intercalation requirements are automatically scheduled to gain EA points and thus are given advantages by the FPAS.

We are also increasingly concerned that the current FPAS promotes a tick-box culture in which substandard engagement is rewarded by points and genuine interest in research is not promoted. For example, recent research has shown that approximately one-third of medical students obtain an intercalated degree [8], but only 16% of these students pursue an academic career [6]. This concept is also reflected when students have their work published while in medical school. The incentive of obtaining EA points by publishing articles compels medical students to submit articles that require less time and effort, such as letters to the editors, compared to the harder alternative of original research publications. As a result, the benefits of original research, such as developing scientific, statistical, and critical appraisal skills, are overshadowed. A study across 7 UK medical schools revealed that only 21% of students who submit articles for publication do so due to having genuine academic interests, whereas 51% of students submit articles purely for career progression [9]. Therefore, it is integral to resist tick-box culture by removing or restructuring EA points and refocusing medical education to encompass clinical academia within its core curriculum.

We are also mindful of the role that the FPAS plays in creating a maldistribution of academically inclined graduates across the United Kingdom. Students with additional degrees and publications receive more EA points and thus rank higher in the FPAS. This allows them to have their preferred choice in deaneries and hospitals prioritized for Foundation Programme allocation [2]. Therefore, more academically inclined, higher-scoring students are recruited into oversubscribed deaneries. In 2019, 11 of the top 20 ranked National Health Service trusts for research were situated in the most competitive deaneries [10]. Consequently, research-minded students are more likely to enter the foundation programs of trusts with more academic opportunities. This perpetuates a cycle of clinical research in these competitive trusts. As a result, a disparity in the advancement of health care may arise across the country, as undersubscribed trusts may fall behind due to a lack of more academically motivated students. These academic hubs across the country are also likely to cultivate competition among students who aim to secure a spot in trusts. This system perpetuates a problematic culture that focuses on unhealthy competition, which is inherent to any point-based ranking system. Conversely, the ideal mentality would be focusing on self-improvement due to having a true interest in medical practice and science.

## The Advantages of EA Points in the FPAS

For many medical students, EAs offer an introductory insight into the field of academic medicine. This involvement is essential for encouraging students, especially due to the downward trend of doctors engaging in research. Moreover, intercalation and publications offer additional benefits at the postgraduate level, such as developing research competencies and promoting the practice of evidence-based medicine [6]. The exclusion of EAs from the FPAS has the potential to discourage students from pursuing academic avenues later on in their careers due to their lack of experience, thereby jeopardizing the future community of clinical academics.

Some studies have reported that EAs in medical school have an unclear predictive effect on successful Foundation Year Program completion compared to decile rankings and Situational Judgement Test scores; hence, their benefit to the FPAS has been questioned [11,12]. However, we believe that the value of EA points is greater than that of their sole contribution to the FPAS and Foundation Year Program.

Undertaking clinical research can benefit students during their medical curriculum. A study revealed that students who completed an intercalation had higher exam results upon resuming their medical degree [13]. This finding was most profound when evaluating the scores of final year students. This improvement may indicate that intercalation leads to the development of better learning techniques, greater analytical and organizational abilities, and enhanced self-directed learning methods. Additionally, intercalated degrees are frequently examined by using essay-based questions, which support the development of critical and divergent thinking as well as scientific writing skills. These skills are valuable to doctors, as they improve clinical communications and reasoning and thus improve patient care. Moreover, obtaining additional degrees provides medical students with the opportunity to work alongside individuals from nonmedical backgrounds, much alike an interdisciplinary team in a clinical setting.

The removal of EA points will also inevitably reduce students’ motivation to publish their work in journals. Publishing articles as a medical student is strongly associated with better future academic achievements. For example, studies have concluded that medical students who have their work published prior to graduating from medical school are almost twice as likely to publish again following graduation [14]. Moreover, studies have also revealed that students who have their work published prior to graduation go on to publish a greater number of papers after graduation and publish papers with higher citation impact [14]. We therefore acknowledge the immense added value of contributing publications while in medical school, given the importance of medical academia to doctors.

EA points can also influence the progression of junior doctors in their training. Specialty training programs are competitive and involve a strict selection process that takes into account academic excellence, extracurricular achievements, and interview performance [15]. Intercalated degrees and publications in an applicant’s portfolio can provide significant evidence of one’s interest in and early commitment to a specialty. However, removing EA points may discourage medical students from obtaining intercalated degrees, thereby resulting in a weaker and less diverse portfolio. Thus, socioeconomically deprived students may end up being disadvantaged later in their career due to being less likely to undertake EA opportunities.

## Moving Forward

EAs add an academic-enriching aspect to medical degrees. Despite their association with systemic inequity, perhaps a preferred solution for encouraging engagement with medical academia should involve widening the participation of disadvantaged students as opposed to removing EAs completely. To encourage more disadvantaged students to intercalate, medical institutions can offer scholarships, subsidize intercalated degree costs, and offer bursaries. A study at the University of Aberdeen identified that early research exposure in medical school in the form of an 8-week program that involved an academic supervisor encouraged intercalation [16]. The results showed that 66% of participants who were undecided on whether to pursue an intercalated degree opted to do so after completing the mentoring program [16]. As such, we believe that launching early, research-based opportunities for socioeconomically disadvantaged students and offering a form of financial support will motivate students in clinical academia and minimize the issues associated with inequality.

We further recommend that medical schools consider Nottingham Medical School’s approach to integrating an intercalated BMedSci Honors Year Project as a standard constituent within 5-year medical courses [17]. This project was conducted over 4 months during the 5-year medical curriculum and provided valuable insight into balancing research while also undertaking clinical responsibilities. This format ensured that all students within the medical school were able to access research opportunities without the common obstacles of financial constraints and limited resources. Often, students from low-income backgrounds are more likely to have part-time jobs, which may limit their ability to pursue research opportunities. Incorporating programs that foster academic skills will ensure that these students will have access to research opportunities.

The promotion of short and time-efficient research opportunities offer an alternative to obtaining additional degrees and promote proactivity in clinical academia. In New Zealand, 75% of students who underwent a 2- to 3-month summer studentship expressed an interest in further research opportunities as a result of their studentship [18]. Similarly, a University of Auckland longitudinal study that investigated summer research studentships revealed that one-third of participating students published at least 1 article with their supervising team within the 10-year follow-up [19]. Positive outcomes in advancing clinical academics were also reported during audits; research electives; and student-led initiatives, such as The Student Audit and Research in Surgery collaborative [18,20].

We would also like to take this opportunity to urge medical journals to adopt a more student-friendly approach. An example of this is the allocation of student-dedicated spaces within journals, such as those in *JMIR Medical Education*, *The Lancet*, and the *Student British Medical Journal* [21]. There have also been an increasing number of student-led peer-reviewed journals that allow students to publish their research [21]. These platforms allow students to familiarize themselves with the process of writing and submitting publications. It also introduces them to the peer-review system, and interested students can even partake in critically appraising submissions. We strongly feel that such initiatives would encourage more medical students to consider publishing their work and promote a genuine interest to contribute to the scientific community. They would also inspire future research and widen student readership.

## Conclusion

The General Medical Council’s “Good Medical Practice” document states that doctors “must be competent in all aspects of work, including management, research and teaching” [22]. We strongly believe that to fulfill the expected, multidimensional qualities of a doctor, it is essential for medical students to have exposure to and experience with academic medicine. As such, while we appreciate the reasoning behind the UKFPO's decision to remove EA points from the FPAS to promote equality among medical students of all socioeconomic backgrounds, reduce the misdistribution of academically inclined graduates, and minimize the degree of damaging competitiveness, we also recognize the multifaceted significance and value of EAs. Consequently, we are concerned with the negative impacts that will result from the removal of EA points.

To minimize the negative outcomes of EAs and maintain their benefits, we urge medical schools to provide greater support nationwide. Studies have reported that as little as 15% of medical students are well informed about research opportunities, intercalating, and publishing [8]. This highlights a need for medical schools to educate students about the benefits of undertaking research opportunities. Such education allows students to make informed choices when pursuing research opportunities, irrespective of EA points. We also hope that medical schools implement more measures for widening the participation of disadvantaged students, especially in research.

To further support students in the absence of EA points, we encourage medical schools to increase the promotion, provision, and accessibility of research-based opportunities in order to produce well-rounded doctors and promote students’ engagement with clinical academia. As such, we propose that EAs should not be removed until the aforementioned measures are defined and in place. We believe that focusing more on promoting clinical research and providing opportunities in academia will turn curious students into inquisitive researchers.

We believe that the removal of EA point scores should take effect only for new cohorts of medical students. Many students who are set to graduate in 2023 are currently in the process of obtaining additional degrees and contributing publications (or have already done so). The exclusion of their hard-earned achievements from contributing to their FPAS score at such late notice is unreasonable. Ultimately, we believe that a delay in the implementation of the UKFPO policy will allow medical schools to become more prepared in supporting its students as well as ensuring that current students are not subject to unexpected, last-minute changes.

In their decision, the UKFPO consulted with representatives from the British Medical Association [3], Medical Schools Council [23], and various other stakeholders. Of concern is the fact that the UKFPO ignored the opinions of the British Medical Association and Medical Schools Council, who also strongly opposed the removal of EA points. In the future, we request that the UKFPO be more receptive to voiced concerns.

Finally, we would like to highlight that in 2015, the UKFPO implemented reforms to the FPAS. Due to these reforms, academic prizes and conference presentations no longer contribute toward EA points [24]. To date, there is no research on the effect of this policy change in terms of the number of prizes received as well as quantity and quality of conference presentations. As such, we urge an investigation into the 2015 policy change, as this may provide insights into the impact of current policy reforms.

## Abbreviations

EA: educational achievement
EPM: Educational Performance Measure
FPAS: Foundation Programme Application System
UKFPO: UK Foundation Programme Office
